# A Manta Ray-Inspired Biosyncretic Robot with Stable Controllability by Dynamic Electric Stimulation

**DOI:** 10.34133/2022/9891380

**Published:** 2022-07-05

**Authors:** Chuang Zhang, Yiwei Zhang, Wenxue Wang, Ning Xi, Lianqing Liu

**Affiliations:** ^1^ State Key Laboratory of Robotics, Shenyang Institute of Automation, Chinese Academy of Sciences, Shenyang 110016, China; ^2^ Institutes for Robotics and Intelligent Manufacturing, Chinese Academy of Sciences, Shenyang 110169, China; ^3^ School of Automation and Electrical Engineering, Shenyang Ligong University, Shenyang 110159, China; ^4^ Emerging Technologies Institute, Department of Industrial & Manufacturing Systems Engineering, University of Hong Kong, Pokfulam, China

## Abstract

Biosyncretic robots, which are new nature-based robots in addition to bionic robots, that utilize biological materials to realize their core function, have been supposed to further promote the progress in robotics. Actuation as the main operation mechanism relates to the robotic overall performance. Therefore, biosyncretic robots actuated by living biological actuators have attracted increasing attention. However, innovative propelling modes and control methods are still necessary for the further development of controllable motion performance of biosyncretic robots. In this work, a muscle tissue-based biosyncretic swimmer with a manta ray-inspired propelling mode has been developed. What is more, to improve the stable controllability of the biosyncretic swimmer, a dynamic control method based on circularly distributed multiple electrodes (CDME) has been proposed. In this method, the direction of the electric field generated by the CDME could be real-time controlled to be parallel with the actuation tissue of the dynamic swimmer. Therefore, the instability of the tissue actuation induced by the dynamic included angle between the tissue axis and electric field direction could be eliminated. Finally, the biosyncretic robot has demonstrated stable, controllable, and effective swimming, by adjusting the electric stimulation pulse direction, amplitude, and frequency. This work may be beneficial for not only the development of biosyncretic robots but also other related studies including bionic design of soft robots and muscle tissue engineering.

## 1. Introduction

Nature has provided great support for the development of robots. On the one hand, bionics is one of the significant robotic research methods, in which the construction and behavior of natural organisms were imitated to improve the kinematic performance and functionality of robots, such as flight [1], walking [2], swimming [3, 4], and adhesion [5]. These current bionic robots composed of artificial materials have made significant progress and effectively promoted robotic development [6]. On the other hand, biosyncretic robots, which take advantage of natural biological materials as robotic core elements, have the potential to further promote the progress in robotics and have attracted increasing attention [7–11]. For example, some microorganism shells have been adopted for microrobots, benefiting from natural precision machining [12, 13]. Moreover, some living cells have been used to realize robotic functions, including sensing [14–16], control [17], and actuation [18], considering their excellent biological performance.

Among these functions, actuation as the main robotic work execution and energy consumption mechanism relates to the robotic overall performance. As previously reported, biological actuators can convert chemical energy into mechanical work at a much higher efficiency than artificial actuators [19, 20]. Biological cells can integrate sensing, control, and actuation functions within a small cell volume of tens of micrometers [18] and generate an actuation force of up to 10 *μ*N [21, 22]. In contrast, the reported smallest artificial motor is up to millimeters and can generate a torque of only approximately 10 *μ*N·m. Moreover, the devices for sensing and control functions increase the overall size of the actuation unit [18]. Additionally, the energy density of carbohydrates for biological actuators can reach 17 MJ/kg, which is much higher than that of current batteries (approximately 1 MJ/kg) for artificial actuators [23]. Therefore, biological materials could effectively improve robotic actuation performance. In particular, biological actuation may be more beneficial for robot miniaturization than traditional electromechanical system actuation.

Because of the potential advantages of biological actuation, different living actuators have been adopted to explore the development of biosyncretic robots [24, 25]. The frequent biological materials used for robotic actuation include movable microorganisms [26], insect dorsal vascular tissues (DV tissues) [23, 27, 28], mammalian cardiomyocytes [29–34], and skeletal muscle cells [35–40]. Among these materials, bacteria and DV tissues can generate an effective actuation force with robustness, but their use is challenging in terms of customizing the actuator size for adaptation to different robots [18]. Although cardiomyocytes can be cultured to form a desired size actuation layer with different amounts of cells, they are difficult to control due to their spontaneous contractility. In contrast, skeletal muscle cells, as the main actuator of mammals, possess the advantages of multiple scales, controllability, and high actuation force [7, 37]. Therefore, they are more suitable for the actuation of biosyncretic robots and may be an ideal candidate for developing more complex robots [41]. The skeletal muscle cells have been used to actuate different robots, including walkers [35, 38, 42], swimmers [36], crawlers [43], and manipulators [37, 44]. In addition, based on the responsibility of the living cells to external stimulation, different control methods for biosyncretic robots have been attempted, such as the most common optical stimulation-based and electric stimulation-based control methods.

As to the optical stimulation method, it involves the use of a focused light spot to stimulate a patch of muscle cells to execute contraction [24, 42, 45]. Due to the controllability of the light spot, the optical stimulation method has the advantage of the high spatiotemporal resolution. However, the muscle cells need to be additionally reprogrammed by optogenetics to achieve photoresponsive feature. In addition, an external device with a specific light source must follow the moving robot in real time to stimulate the target living actuator. Therefore, these factors may increase the system complexity and restrict the robot’s kinematic dexterity to some degree [18]. As to the electric stimulation method, it usually uses a pair of fixed electrodes to stimulate muscle cells or tissues within the electric field area. This approach can realize noncontacting control of the actuation frequency and amplitude of muscle cells or tissues with a simple system. However, the high electric potential may induce medium electrolysis and electrochemical cell damage [18, 46]. Moreover, for mobile biosyncretic robots, the included angle between the cell axis and electric field generated from a pair of fixed electrodes changes with the robot’s dynamic movement. According to our previous work [43], the changing electric field direction related to the cell axis induces different contractility of the cells. Therefore, it may be difficult for a pair of fixed electrodes to realize the stable controllable motion of biosyncretic robots.

In this paper, to promote the stable controllable motion performance of the biosyncretic robots, a bionic swimmer actuated by a cultured skeletal muscle tissue and controlled by circularly distributed multiple electrodes (CDME) was studied. The robot structure inspired by a manta ray was designed to realize effective propelling actuated by only one muscle tissue. A direction-controllable electric field generated from the CDME was used to stimulate the muscle actuation tissue of the robot. This could ensure the stable controllability of the muscle actuation and robot swim by maintaining a real-time parallel between the actuation tissue and the stimulation electric field. In addition, it has been verified in our previous work that the electric field from the CDME was less harmful to the medium and cells than those of the traditional electrodes [43]. The major structure of the swimmer was fabricated with polydimethylsiloxane (PDMS) casting. For the convenient assembly of the living actuator and robot structure, circular muscle tissues were manufactured and cultured. Additionally, to obtain the circular muscle tissues with effective contractility, a rotational electric stimulation from the CDME has been used to realize uniform induction of the myoblasts to differentiate into myotubes. To control the robot to swim at a desired speed, the contractility of the muscle tissue was measured before assembly with the swimmer structure. In addition, the relationship between the motion performance of the swimmer and the contractility of the actuation tissue was analyzed with the simulation method. Finally, the biosyncretic swimmer has demonstrated effective swimming with stable controllability and verified the validity of the proposed bionic design and CDME-based control methods. This work may be beneficial for not only the further development of biosyncretic robots but also other related fields such as the bionic design of soft robots and muscle tissue engineering.

## 2. Materials and Methods

### 2.1. Overall Design of the Biosyncretic Swimmer

**Figure 1:**
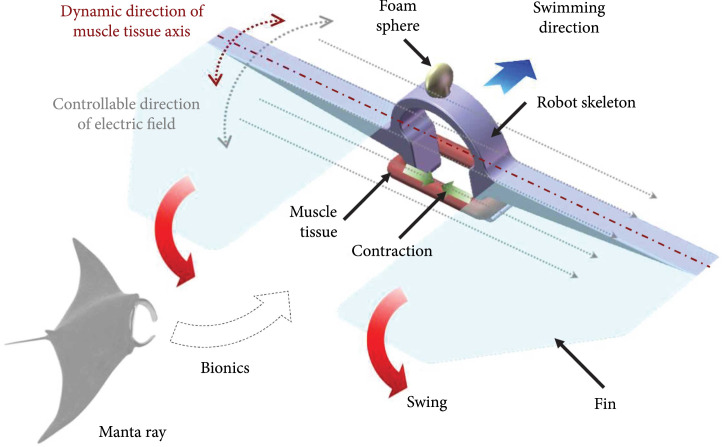
Schematic diagram of the proposed biosyncretic swimmer.

To improve the stable controllable motion performance of biosyncretic robots, a manta ray-inspired miniature bionic swimmer actuated by a cultured muscle tissue and controlled by a direction-controllable electric field induced with CDME was proposed (Figure 1). The overall structure of the biosyncretic swimmer was designed with the commercial software SolidWorks. This swimmer was composed of a skeleton structure, two fins, a living actuator, and a foam balance microsphere. The skeleton structure was fabricated by PDMS casting. The fins were made of polyimide (PI) film. The living actuator was produced by 3D culturing of C2C12 cells. The control system of the biosyncretic robot consists of 8 circularly distributed platinum electrodes around the swimmer. Each electrode is connected to an independent channel of an electric impulse stimulator. According to our previous work [43], the electric field with the desired direction could be generated by separately controlling the potential of each electrode. Therefore, the electric pulse of real-time parallel to the muscle actuation tissue axis could be maintained during the robot’s dynamic swimming. And then, the instability of the tissue actuation induced by the dynamic unparallel relation between the tissue axis and electric field direction could be eliminated. Hence, the biosyncretic swimmer could be controlled to stably swim with a desired speed.

### 2.2. Design and Fabrication of the Robot Skeleton

The robot skeleton was designed according to the structure of a manta ray. The PDMS structure was fabricated through a traditional casting process, as described in our previous report [47]. First, the physical dimension of the PDMS structure was designed with SolidWorks. Then, the designed 3D model was imported into the program software of a minitype miller (ROLAND EGX-400, Japan) to fabricate the polymethyl methacrylate (PMMA) negative mold of the PDMS structure. After that, uncured PDMS at a proportion of 10: 1 was added to the PMMA mold followed by baking at 80°C for 4 hours in a constant temperature heating box (Heratherm OGS60, Thermo). Finally, the cured PDMS structure was carefully lifted from the mold and assembled with the cultured living tissue, PI fins, and foam microsphere.

### 2.3. Culture of the Living Actuator

The muscle actuation tissue used for the proposed swimmer was fabricated with a circular mold with rotary electric stimulation similar to our previous work [47]. The core living material was C2C12 myoblasts (American Type Culture Collection, Manassas, VA, USA). The other reagents in the actuation tissues were Matrigel (Solarbio, Beijing, China), fibrinogen (Sigma Aldrich, St. Louis, MO, USA), and thrombin (Sigma Aldrich, St. Louis, MO, USA) [43]. In the process of tissue culture, two kinds of medium were prepared for the growth and differentiation of C2C12 cells. The growth medium (GM) was composed of Dulbecco’s modified Eagle’s medium (DMEM, 89% High glucose, Gibco), fetal bovine serum (10%, Gibco), and penicillin-streptomycin (1%, Gibco) and was used for the proliferation of the myoblasts in 3D gelatin tissues. The differentiation medium (DM) consisted of DMEM (97%), horse serum (2%, Gibco), and penicillin-streptomycin (1%) and was used for differentiation induction of the myoblasts into contractile myotubes.

**Figure 2:**
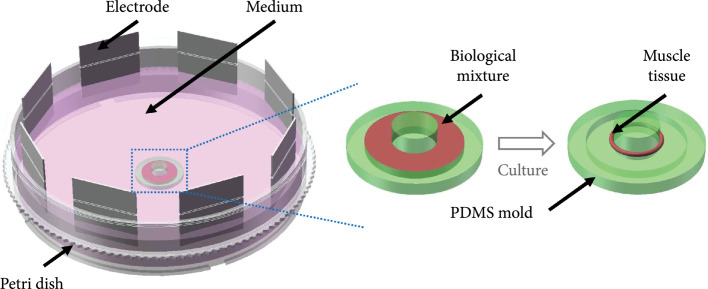
Schematic diagram of 3D muscle tissue fabrication.

The fabrication process of the 3D muscle tissues was as follows (Figure 2): (1) The biological mixture, including the cells, Matrigel, fibrinogen, thrombin, and GM, was injected into a PDMS mold, which was fabricated by the same PDMS casting method for the robot skeleton structure and fixed in a 100 mm petri dish. (2) The petri dish was placed in a cell incubator at 37°C and 5% CO_2_. (3) One hour later, the GM was injected into the dish to submerge the PDMS mold. (4) The GM was removed after culturing for 2 days; then, the 3D tissues were washed 3 times with phosphate buffered saline (PBS, HyClone); after that, the DM was added to the dish to replace the GM. (5) Two days later, rotary electric stimulation was applied to the muscle tissues with 8 circular distributed platinum electrodes after the DM was renewed. The size of the electrodes was 20 mm in length, 20 mm in width, and 0.2 mm in thickness. Each electrode was connected to an independent connector of an electric stimulator (Master-9, AMPI). The electric pulse was set as 1 Hz frequency, 10 V amplitude, and 10 ms pulse width. The step angle of the electric field direction was 45°, and the stimulation time in each direction during the rotary stimulation was 30 s. The electric stimulation was sustained for 24 hours with alternating 2 min on and 10 min off. (6) Eight days later, the muscle actuation tissues were peeled off from the PDMS molds for the subsequent contractility measurement and assembly with the swimmer body.

### 2.4. Elasticity Measurement of the PDMS Structure

For the mechanical property measurement of the PDMS structure, a Dimension Icon AFM system (Bruker, Santa Barbara, CA, USA) was used in this work. Fully taking the liquid working environment of the swimmer into account, the PDMS structure was measured in the same medium to ensure measurement accuracy. The probe used in this work was an MLCT-type-C (Bruker), and the nominal spring constant of the selected cantilever was 0.01 N/m. Before the measurement, the PDMS structure was fixed in a 60 mm petri dish. Then, the petri dish was placed on the objective table of the AFM. In the measurement process, first, the AFM tip was controlled to go down into the DM in the petri dish and touch the substrate of the dish to obtain a force curve for calibration of the cantilever deflection sensitivity. After that, the accurate spring constant of the used cantilever was acquired using the thermal tune function of the AFM system. Next, 100 force curves were obtained at random different points on the PDMS structure surface (10 curves for each point). Finally, based on the obtained force curves, the Hertz model was used to calculate the Young’s modulus of the PDMS structure, as in previous reports [48, 49].

### 2.5. Contractility Measurement of the Living Actuator

**Figure 3:**
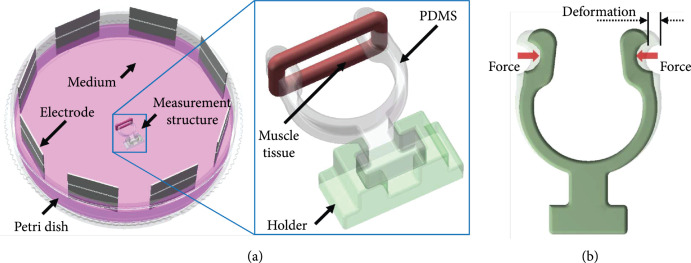
Schematic diagram of muscle tissue measurement. (a) Schematic diagram of the PDMS measurement structure actuated by a muscle tissue and (b) schematic diagram of the deformation simulation of the PDMS structure under an actuation force.

The contractility of the tissue under an electric pulse stimulation with a parallel direction to the tissue axis, different frequencies, and amplitudes was measured. In the process, the tissue to be measured was assembled with a fabricated PDMS measurement structure, which was fabricated by the same PDMS casting method for the robot skeleton structure (Figure 3(a)). The PDMS structure deformed under the actuation of the muscle tissue controlled by the electric stimulation, and the structure deformation was recorded with a commercial microscope (Nikon, Ti-E, Japan) and analyzed with the previous MATLAB procedure [49]. Then, based on the deformation results, the contractility of the measured tissue could be obtained by COMSOL simulation with the mechanical properties and geometric dimensions of the PDMS measurement structure (Figure 3(b)) [47].

### 2.6. Simulation of the Electric Field and Fluid-Solid Coupling

The electric fields generated by a pair of electrodes and the proposed CDME were simulated with the COMSOL software. In the simulation process, the 3D models of the electrodes, medium, and petri dish were obtained with SolidWorks software at first. Then, the 3D models were imported into the simulation software. The electrical resistivities of the materials for the simulation models of platinum electrodes, medium, and petri dish were, respectively, set as $2.22\times 10^{-7}\;\Omega \cdot \text{m}$, $6.67\times 10^{-2}\;\Omega \cdot \text{m}$, and $10^{16}\;\Omega \cdot \text{m}$, according to their material characteristics. Finally, the electric fields of the different stimulation devices of a pair of electrodes and CDME were stimulated and analyzed by applying different voltages on the electrodes.

According to the previous related reports [50–52], the fluid-solid coupling module of COMSOL software was used to simulate the actuation of the muscle tissue on the soft structures in liquid, including the force measurement structure and robot structure. In the simulation process, at first, the 3D models of the soft structures were built with SolidWorks software based on the real dimensions and were imported into the simulation software. Then, a rectangular fluid domain of 80 mm×80 mm×40 mm was created to form the liquid environment. After that, the 3D models of the soft structures were placed in the center of the fluid domain. The model materials of the force measurement structure and robot skeleton were set as PDMS. And the model material of the fins was set as PI. The corresponding material parameters were set based on the measured values with AFM in this work. Finally, the deformation of the measurement structure and the swimming of the robot were simulated and analyzed under the actuations with different forces and frequencies.

### 2.7. Swimming Control of the Biosyncretic Swimmer

**Figure 4:**
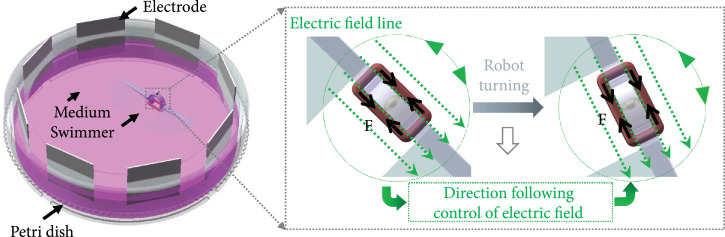
Schematic diagram of the biosyncretic robot swimming dynamic control method with the CDME.

To demonstrate the stable controllable motion of the proposed biosyncretic robot, the swimmer was stimulated to swim at different speeds in a 100 mm petri dish (Figure 4). Similar to the stimulation system used for muscle tissue culture, 8 platinum electrodes were circularly distributed around the petri dish. Each electrode was connected to an independent channel of the electric stimulator. The potential of each channel can be adjusted independently. Thereby, the direction of the electric field could be controlled in real time to maintain the parallel relation to the muscle actuation tissue.

Based on the contractility measurement results of the living tissue and the simulation results of the robot swimming, the electric pulse with different parameters, including amplitude and frequency, was applied to the biosyncretic robot. And the direction of the stimulation electric field could be controlled by adjusting the potentials of the 8 electrodes. In case of the electric field direction was controlled to be real-time parallel to the actuation tissue, the muscle tissue could execute stable contractility and actuate the robot to swim with a stable speed affected by only the stimulation pulse amplitude and frequency, but not the dynamic robot posture.

### 2.8. Statistical Analysis

In this work, a written MATLAB program was used to analyze the PDMS measurement structure deformation and the robot motion performance by converting the dynamic videos to continuous static pictures [47]. The sample sizes of the deformation measurement of the PDMS structure and the speed measurement of the swimmer under different electrical stimulation were 5. And these data were demonstrated with box plot. The sample size of the young’s modulus measurement of the PDMS structure was 100. And this data was demonstrated with a Gaussian fitting plot.

## 3. Results and Discussion

### 3.1. Simulation of the Electrical Characteristics of Different Stimulation Devices

**Figure 5:**
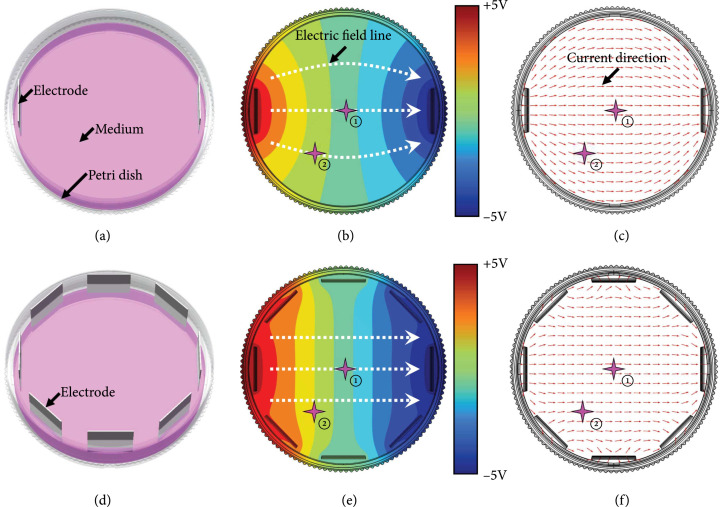
Simulation results of the electrical characteristics of different stimulation devices. (a, d) The 3D models of the devices based on a pair of electrodes and CDME; (b, c) the simulation results of the electric field distribution and current direction generated by a pair of electrodes; and (e, f) the simulation results of the electric field distribution and current direction generated by the CDME.

To demonstrate the advantages of the proposed CDME for the culture of muscle tissues and control of the biosyncretic robots, the electrical characteristics of different stimulation devices based on CDME and traditional parallel electrodes have been simulated and analyzed. As to the stimulation device consisted of a pair of parallel electrodes, the potential of one electrode was set as +5 V, and that of the other was set as -5 V, to form a maximum electric potential difference of 10 V in the petri dish (Figure 5(a)). Meanwhile, for the stimulation device based on CDME, the potentials of a pair of opposite electrodes were set as +5 V and -5 V, respectively. These were similar to that for the above device based on parallel electrodes. What is more, to obtain a parallel electric field, the potentials of the other electrodes were set to the corresponding values, which were proportional to the perpendicular distance between the electrode and the centerline of the petri dish (Figure 5(d)).

The simulation results showed that both the electric field line and the current direction of the CDME possessed better directivity than those of a pair of electrodes (Figure 5). Additionally, the electric field direction of the CDME could be changed by controlling the potential of each electrode, while the field direction of a pair of electrodes was fixed. The electrical characteristics of the CDME may be more suitable for the culture of circular muscle tissues and the control of the dynamic robots than those of a pair of electrodes. For example, on the one hand, to realize the uniform differentiation of the C2C12 cells in the circular tissue, all the cells should be stimulated. However, the fixed electric field from a pair of electrodes could not execute a rotary stimulation, which could be implemented with CDME. On the other hand, to realize stable control of a dynamic robot, the electric field direction should be real-time parallel to the muscle tissue axis [43]. For this, the CDME could generate a direction-controllable electric field. Also, the electric field direction of different locations in the electric field region generated by CDME is almost the same (such as ① and ② in Figures 5(e) and 5(f)). Therefore, the parallel relationship between the field direction and the tissue axis could be maintained in real time with CDME. However, for a pair of electrodes, neither the parallel relationship could be maintained nor the electric field directions were consistent at different locations (such as ① and ② in Figures 5(b) and 5(c)). Based on the above analysis, the stimulation device based on the CDME, but not the traditional stimulation device with a pair of electrodes, could be competent for the circular tissue culture and mobile robot control.

### 3.2. Electroresponsive Behavior of the Living Actuation Tissue

**Figure 6:**
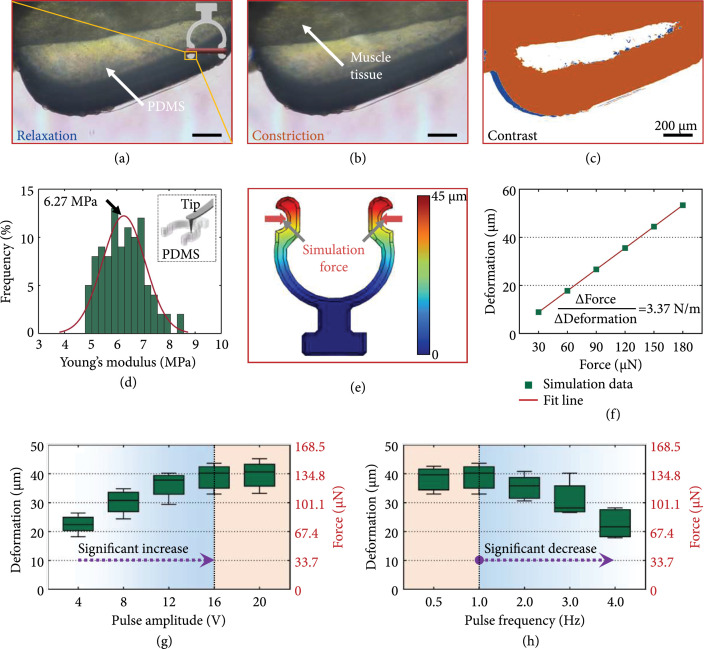
Contractility measurement of the muscle tissue. (a) and (b) One end of the PDMS measurement structure, when the muscle tissue is relaxed and constricted, respectively; (c) the comparison of the PDMS structure with and without the contraction of the muscle tissue; (d) statistical result of Young’s modulus of the measurement structure; (e) simulation deformation of the measurement structure under simulation force; (f) relationship between simulation deformation of the measurement structure and actuation force; (g) relationship between muscle tissue actuation and stimulation pulse amplitude; (h) relationship between muscle tissue actuation and stimulation pulse frequency.

The 3D muscle tissue actuators were fabricated through the culture method described above. To control the swimmer by stimulating the living actuator, the electroresponsive behavior of the living actuator was measured with the fabricated contractility measurement structure (Figures 6(a)–6(c), Movie S1). When the muscle tissue was stimulated with the same frequency (1 Hz) and different pulse amplitudes (from 4 V to 20 V), the measurement structure was deformed with different displacements. As shown in Figure 6(g), the amplitude of the structure end increased with the pulse amplitude when the pulse amplitude was between 4 V and 16 V, whereas the deformation of the measurement structure no longer increased observably when the pulse amplitude changed from 16 V to 20 V. This result was similar to those of previous reports [37, 47] and could be attributed to the recruitment phenomenon. This means that the number of working myofibers increased with the stimulation voltage until all the myofibers were activated.

In addition, as shown in Figure 6(h), when the muscle tissue was stimulated with the same pulse amplitude (16 V) and different frequencies (from 0.5 Hz to 4 Hz), the measurement structure exhibited different deformations. When the stimulation frequencies were 0.5 Hz and 1 Hz, the deformations were similar. However, the measurement structure showed decreasing amplitude with increasing stimulation frequency from 1 Hz to 4 Hz. This phenomenon was in line with the related work [41] and may be explained below. When the tissue was stimulated with a low-frequency pulse, the recovery time of the tissue from the contracted state to the relaxed state was less than the pulse separation. Therefore, the tissue had similar dynamic contraction amplitudes under low-frequency stimulation. However, with the increase in the stimulation frequency, the pulse separation became insufficient for a complete recovery of the tissue. Hence, the tissue stimulated with high frequency demonstrated an incomplete dynamic amplitude.

After that, the mechanical properties of the PDMS structure were measured with the AFM to obtain the contraction force of the tissues based on the deformation amplitude data measured above. The results showed that Young’s modulus was 6.27±0.82 MPa (Figure 6(d)), which was in line with the related report [53]. In the simulation process, the 3D model of the measurement structure with the real dimensions was created and simulated with COMSOL software. The Young’s modulus and Poisson’s ratio of the simulation structure material were set to 6.27 MPa and 0.495 [54]. Then, the elasticity coefficient of the measurement structure could be obtained by simulating the different deformations of the PDMS structure under different simulation forces (Figures 6(e) and 6(f)). The results showed that the relationship between the measurement structure deformation and actuation force was linear. The elasticity coefficient of the measurement structure was 3.37 N/m (Figure 6(f)). Thereby, the contractility of the measured living actuation tissue was calculated with the obtained elasticity coefficient and deformation data of the measurement structure (Figures 6(g) and 6(h)). The maximal contractile force of the living actuation tissue fabricated in this work was 152.32 *μ*N, which was in line with related reports [18, 55].

### 3.3. Swimming Simulation of the Biosyncretic Swimmer

**Figure 7:**
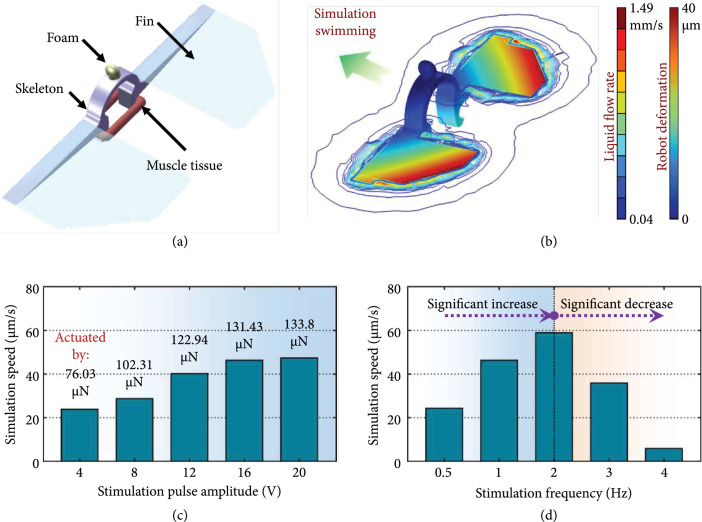
Swimming simulation of the biosyncretic swimmer. (a) 3D model of the proposed biosyncretic swimmer; (b) representative simulation result of the robot swimming in liquid; (c) simulation result of the robot speed under the actuation forces induced by different stimulation amplitudes and fixed frequency of 1 Hz; (d) simulation result of the robot speed under the actuation forces induced by different stimulation frequencies and fixed amplitude of 16 V.

To control the biosyncretic robot to swim at a desired speed, the relationship between the swimming speed and the tissue contractility has been simulated with COMSOL software. According to the simulation method introduced in the previous section, the robot 3D model was built with the real dimensions (Figure 7(a)). Based on the AFM measurement results and related reports [54], the Young’s modulus and Poisson’s ratio of the robot skeleton material (PDMS) were set to 6.27 MPa and 0.495, which were the same as those of the force measurement structure. And those of the fins material (PI) were set to 2.10 Gpa and 0.34 [56, 57].

According to the contractility measurement results of the living actuation tissue stimulated by different pulse amplitudes and frequencies (Figures 6(g) and 6(h)), different actuation forces and frequencies were used in the robot swimming simulation (Figure 7(b)). The result showed that when the actuation frequency was set as 1 Hz, the simulation speed of the swimmer increased with the actuation force, which was obtained under different stimulation pulse amplitudes (from 4 V to 20 V). And the changing tendency of the speed with the increasing pulse amplitude was in line with the measurement result of the tissue contractility (Figures 6(g) and 7(c)). In addition, when the swimmer was actuated with the forces induced by the same stimulation pulse amplitude (16 V) and different frequencies (from 0.5 Hz to 4 Hz), the simulation speed increased at first and then decreased with the stimulation frequency (Figure 7(d)). This phenomenon may be attributed to the swimming speed being affected by both the fins’ swinging deformation and frequency. Although the flapping frequency of the fins increased with the stimulation frequency, the flapping deformation gradually decreased due to the declining actuation force (Figure 6(h)). Therefore, the comprehensive propelling performance showed a trend of first increasing and then decreasing with the increasing actuation frequency.

### 3.4. Actuation and Control of the Biosyncretic Swimmer

According to the overall design, the proposed biosyncretic robot consisted of a swimmer and a CDME-based external control system. The swimmer was assembled with a casted PDMS skeleton structure, two PI fins, a cultured muscle tissue actuator, and a foam balance microsphere. The two fins were fixed on the robot skeleton structure with PDMS. The muscle tissue was assembled on the anchor structure of the swimmer. Because the densities of the PDMS, PI, and muscle tissue were higher than that of the culture medium, the foam microsphere was fixed at the top center of the robot skeleton to balance their gravity.

**Figure 8:**
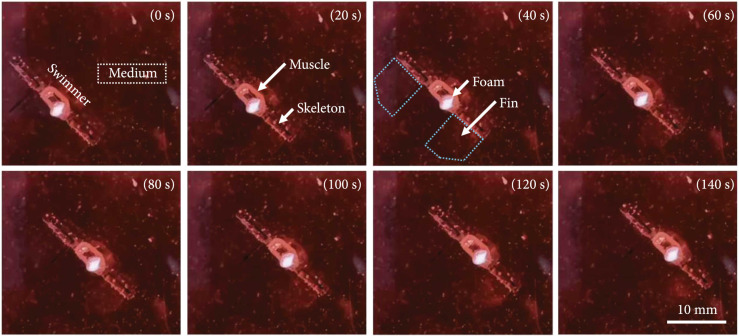
Representative swimming record of the biosyncretic robot stimulated by a pulse with the following parameters: parallel direction to the muscle tissue, 2 Hz frequency, and 16 V amplitude.

**Figure 9:**
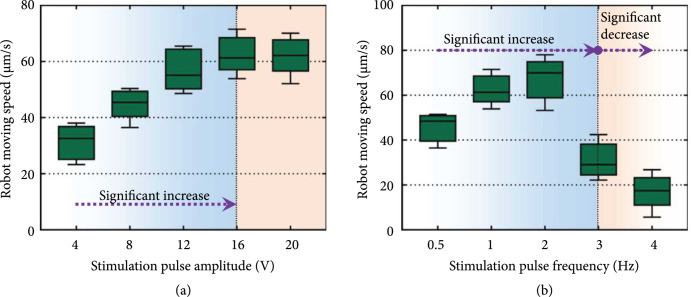
Actuation control of the biosyncretic swimmer. (a) Relationship between the robot swimming speed and stimulation pulse amplitude and (b) relationship between the robot swimming speed and stimulation pulse frequency.

To demonstrate the controllable motion of the developed biosyncretic swimmer, electric stimulation signals in the direction of the actuation tissue and with different amplitudes and frequencies were used to stimulate the tissue of the robot. When an electric stimulation pulse was applied to the living tissue through the CDME, the fins were actuated to execute up and down swinging to propel the swimmer (Figure 8, Movie S2). According to the results of the actuation tissue contractility measurement and robot swimming simulation, the amplitude and frequency of the electric pulse were adjusted to control the speed of the swimmer. The results showed that when the stimulation frequency was fixed at 1 Hz, the swimming speed presented the tendency of first increasing and then remaining invariant with increasing pulse amplitude. As shown in Figure 9(a), when the stimulation voltage was less than 16 V, the swimming speed increased with the pulse amplitude, and the increasing rate of the speed gradually decreased. When the stimulation voltage increased from 16 V to 20 V, no obvious speed change was observed. This phenomenon was in line with the results of the tissue contractility measurement and the robot speed simulation under different pulse amplitudes (Figures 6(g) and 7(c)) and could be attributed to the recruitment phenomenon of actuation tissue under increasing pulse amplitude, which has been discussed in the Section of 3.2.

In addition, when the pulse amplitude was fixed at 16 V and the frequency was less than 2 Hz, the robot’s swimming speed increased with the stimulation frequency. However, the speed obviously decreased when the stimulation frequency changed from 2 Hz to 4 Hz (Figure 9(b)). This phenomenon was in line with the simulation result of the robot swimming actuated with different actuation frequencies (Figure 7(d)) and could be attributed to the changing combination propelling performance of the fins’ swinging deformation and frequency under the increasing pulse frequency, which has been discussed in the Section of 3.3.

Although there was some error between the experiment and simulation of the robot swimming, the changing tendencies of the swimmer’s moving speed under the electric stimulations with different pulse amplitudes and frequencies (Figure 9) were in line with the corresponding simulation results shown in Figure 7. This error could be attributed to the manufacturing error of the robot, measurement error of the living tissue, the disturbance of the dynamic liquid, and so on. And these potential error factors would be considered in the theoretical model of the swimmer in further work, to further enhance the stable controllability of the biosyncretic robots.

**Figure 10:**
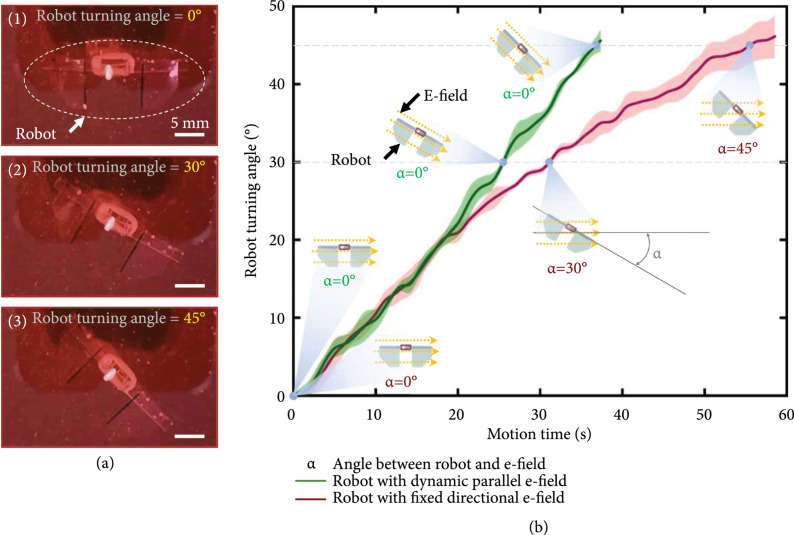
Turning control of the asymmetrical biosyncretic swimmer. (a) Representative turning record of the biosyncretic robot and (b) relationship between the turning angle and motion time of the asymmetrical biosyncretic swimmers, respectively, stimulated with fixed directional electrical field (red line) and dynamic parallel electrical field (green line), under the same stimulation frequency and amplitude (2 Hz and 16 V).

What is more, to further demonstrate the advantages of the proposed robot control method based on CDME, an asymmetric robot was fabricated by fixing one of the two fins of the swimmer with glue, to perform turning swimming under the actuation of living tissue (Figure 10(a)). In the process of the experiment, the asymmetric swimmer was stimulated by different electric fields with fixed direction and dynamic direction, respectively. When the swimmer was stimulated by an electric pulse with 2 Hz frequency and 16 V amplitude in a fixed direction, the swimmer showed a turning motion with changing angular speed (red line in Figure 10(b)). This phenomenon could be attributed to that the contractility of the tissue decreased with the increasing included angle between the stimulation electric field and the axis of the actuation tissue, during the turning motion of the swimmer. This result was in line with our previous report that the muscle tissue had different forces under the electric fields with different included angles with the tissue [43].

Whereas, when the swimmer is under the electric stimulation with the same pulse parameters used above and in a real-time parallel direction to the muscle tissue axis, the swimmer could execute a uniform turning motion (green line in Figure 10(b)). This result was attributed to the direction controllability of the electric field from the CDME, through dynamically controlling the potential of each electrode [47]. Due to the electric field direction of the pulse could be maintained parallel to the tissue axis, there was no influence on the tissue contractility from the dynamic turning motion of the robot. Therefore, the actuation tissue controlled by the proposed method based on CDME could execute stable controllable contractility during the dynamic swimming of the robot and actuate the robot to swim with a stable controllable speed, which was only controlled by the electric pulse parameters, but not affected by the dynamic posture of the swimmer.

## 4. Conclusion

In this work, to advance the controllable motion performance of biosyncretic robots, a muscle tissue-based swimmer with a bionic propelling mode and dynamic control method has been developed, where the bionic propelling inspired by a manta ray was designed to realize effective propelling of the biosyncretic robot actuated by only one actuation tissue. The dynamic control was executed by controlling the direction-controllable stimulation electric field of the CDME to maintain the real-time parallel with the robot actuation tissue and then to ensure stable contractility of the living actuation tissue. In addition, the circular muscle tissues were cultured with the rotary electric stimulation from the CDME, which was verified to be beneficial for cell differentiation, to enhance the actuation force of the living tissues of the robot. Finally, the developed biosyncretic robot has demonstrated stable controllable swimming under the proposed control method with dynamic electric stimulation.

Although the biosyncretic robot developed in this work has realized effective and stable controllable motion, lots of essential works remain to be carried out. For example, the dimensions of the fabricated swimmer were centimeter-level, which may be too large for in vivo drug transport. Therefore, miniaturization technologies, such as 3D printing and flexible manipulation for micro biological structures [58, 59], are necessary for the development of biosyncretic microrobots for clinical applications. Furthermore, most of the existing biosyncretic robots only rely on external artificial stimulations to realize controllable motion and lack autonomy. Therefore, the sensing and control methods based on living cells could be adopted into the biosyncretic robots actuated by muscle cells to realize autonomic motion through responding to environmental information. Nevertheless, this paper is beneficial for the development of biosyncretic robots to some extent. Moreover, the bionic approach used in this work could also be a potential reference for the development of electromechanical system-based soft robots. And the culture method of muscle tissues with a circular mold and rotary electric stimulation may be useful for the study of tissue engineering.

## Data Availability

All data needed to evaluate the conclusions in the paper are present in the paper and/or the Supplementary Materials. Additional data related to this paper may be requested from the authors.

## Supplementary Materials

Supplementary Materials Movie S1: deformation of the force measurement structure actuated by a living tissue, which was stimulated by a parallel electric field with the pulse parameters of 1 Hz stimulation frequency and 20 V voltage amplitude. Movie S2: moving of the biosyncretic swimmer stimulated by a pulse with the parameters: parallel direction to the muscle tissue, 2 Hz frequency, and 16 V amplitude.
